# Computational approaches for inferring the functions of intrinsically disordered proteins

**DOI:** 10.3389/fmolb.2015.00045

**Published:** 2015-08-05

**Authors:** Mihaly Varadi, Wim Vranken, Mainak Guharoy, Peter Tompa

**Affiliations:** ^1^Flemish Institute of BiotechnologyBrussels, Belgium; ^2^Department of Structural Biology, VIB, Vrije Universiteit BrusselsBrussels, Belgium; ^3^ULB-VUB - Interuniversity Institute of Bioinformatics in Brussels (IB)^2^Brussels, Belgium

**Keywords:** intrinsically disordered proteins, IDP ensembles, IDP function, disorder prediction, protein ensemble database

## Abstract

Intrinsically disordered proteins (IDPs) are ubiquitously involved in cellular processes and often implicated in human pathological conditions. The critical biological roles of these proteins, despite not adopting a well-defined fold, encouraged structural biologists to revisit their views on the protein structure-function paradigm. Unfortunately, investigating the characteristics and describing the structural behavior of IDPs is far from trivial, and inferring the function(s) of a disordered protein region remains a major challenge. Computational methods have proven particularly relevant for studying IDPs: on the sequence level their dependence on distinct characteristics determined by the local amino acid context makes sequence-based prediction algorithms viable and reliable tools for large scale analyses, while on the structure level the *in silico* integration of fundamentally different experimental data types is essential to describe the behavior of a flexible protein chain. Here, we offer an overview of the latest developments and computational techniques that aim to uncover how protein function is connected to intrinsic disorder.

## Introduction

The traditional goal of protein structural biology is to relate the well-defined three-dimensional structure(s) of a protein to its biological function. This structure-function paradigm continues to facilitate many important discoveries, but has largely ignored the possible roles of conformational flexibility on function (Forman-Kay and Mittag, 2013). Yet, in the recent years it became apparent that structural disorder is ubiquitously present in diverse cellular processes, and has a particularly prominent role in regulation and signaling events occurring in the complex cellular environment (Tompa et al., 2006; Dunker et al., 2015). Proteins or protein regions that are enriched in conformational flexibility are referred to as intrinsically disordered proteins (IDPs) or protein regions (IDRs) (Dyson and Wright, 2005; Fink, 2005; Tompa, 2005). IDPs and IDRs lack a well-defined, stable three-dimensional fold, and therefore they populate ensembles of dynamically exchanging conformations, separated by low energy barriers. This dynamic behavior challenges the traditional structure-function paradigm (Wright and Dyson, 1999; Chouard, 2011), since it is far from trivial to describe the structural behavior of proteins that adopt such an extensive range of conformations, let alone infer their biological role (Tompa, 2011). Even though intrinsic disorder is occurring ubiquitously—more than 30% of the proteins in the known eukaryotic proteomes have disordered segments of 30 or more consecutive disordered residues (Dunker et al., 2000)—we are only beginning to understand how protein function arises from the disordered state (Tompa, 2011). Here, we provide an overview of the recent developments in terms of computational methods and data resources that facilitate the understanding of intrinsic disorder and its connection to protein function.

## Functional consequences of intrinsic disorder

IDPs and IDRs can be viewed as having complementary functions to those of their folded counterparts. While the latter are often involved in enzymatic activities, molecular transportation or binding short peptides and small molecules, IDPs are mainly involved in signaling, regulation and enzymatic activity inhibition (Xie et al., 2007; Dunker et al., 2015), for example in cell cycle regulation (Yoon et al., 2012), cell division and differentiation (Ward et al., 2004; Xie et al., 2007).

There are a number of possible ways in which an IDP/IDR can realize its function. In perhaps the simplest scenarios, they serve as entropic chains, effectively influencing the orientation and distance between folded domains (Chong et al., 2010), and organizing the super-tertiary structure of the protein (Tompa, 2012). In some cases they are entropic springs or even timers, where the length and flexibility of the linker can determine stochastically how often two folded domains may encounter each other (Bentrop et al., 2001; Smagghe et al., 2010).

Another important role of conformational flexibility is in binding protein or nucleic acid partners. IDPs excel in establishing specific, but transient interactions (Dunker et al., 1998). From an energetic point of view, the reason behind their weaker binding affinities is that the entropic cost of stabilizing a single conformation from the dynamic ensemble that the IDP/IDR is sampling is relatively high (Dyson and Wright, 2005). However, in some cases the fine-tuning of favorable interactions is known to yield surprisingly strong affinities (Ferreon et al., 2013; Follis et al., 2013). An additional advantage of the high degree of conformational freedom is that an IDR can bind very diverse partners because it can easily adopt different conformations (Wang et al., 2011; Hsu et al., 2013) and is often enriched in short binding- and recognition motifs. It is therefore no surprise that IDPs are often hub- (Kim et al., 2008) or scaffold proteins (Dyson and Wright, 2005; Kim et al., 2008; Mittag et al., 2010a) that play essential roles in the cell by integrating signals (Lobley et al., 2007), so increasing the complexity of cellular networks (Dunker et al., 2005; Oldfield et al., 2008). Consequently, IDPs are often implicated in pathological conditions where loss of regulation is the major issue, such as different types of cancer (Andresen et al., 2012). Their involvement in diseases has recently turned IDPs into potential drug targets by either targeting the IDP, or its protein-protein interactions (Funk and Galloway, 1998; Metallo, 2010; Rezaei-Ghaleh et al., 2012).

Conformational flexibility implies high accessibility for potential binding partners and/or enzymes. Consequently, post-translational modification (PTM) sites are often found to be enriched in intrinsically disordered regions (Iakoucheva et al., 2004), with especially phosphorylation sites being prevalent (Gao et al., 2010). While IDPs often go through disorder-to-order transitions upon binding to their partners (Mohan et al., 2006; Wright and Dyson, 2009), in many cases they remain partially or fully flexible in their bound state, forming fuzzy complexes (Tompa and Fuxreiter, 2008; Fuxreiter and Tompa, 2012). One of the advantages of this fuzziness is that PTM sites within the chain can remain relatively accessible, allowing easier regulation of the IDP by modification enzymes (Mittag et al., 2010a). Such regulation by PTM sites is not limited to activation/deactivation of the protein; the modification of the surface of the IDR may also be the prerequisite of binding to a different partner (Oldfield et al., 2008), or even to the same partner, but with increased affinity (Mittag et al., 2010b).

However, intrinsic disorder also has a dark side. In particular, the amino acid compositional bias of IDPs coupled with relatively high propensities to form β-sheets and turns leads to elevated aggregation potentials, and the formation of amyloid-type beta-structures (Levine et al., 2015). Indeed, IDPs have been implicated in aggregation-based diseases, such as Alzheimer's and Parkinson's (Huang and Stultz, 2009; Uversky, 2010).

## Sequence-based investigation of IDPs

There are a number of experimental techniques currently available for identifying and characterizing intrinsic disorder, such as circular dichroism (CD) (Weinreb et al., 1996), protease digestion (Johnson et al., 2012), Förster resonance energy transfer (FRET) (Haas, 2012), Electron Paramagnetic Resonance (EPR) spectroscopy (Drescher, 2012), small-angle X-ray and neutron scattering (SAXS and SANS) (Bernado and Svergun, 2012; Gabel, 2012) and nuclear magnetic resonance spectroscopy (NMR) (Kosol et al., 2013; Konrat, 2014). For initial and for high-throughput investigations, computational methods are however a very popular choice (Ward et al., 2004; Ishida and Kinoshita, 2007). Intrinsic disorder is associated with distinct sequence characteristics; IDPs/IDRs are enriched in “disorder promoting” amino acids, such as charged or polar residues, glycines and prolines, while hydrophobic residues are underrepresented (Uversky et al., 2000). Their conformational flexibility also implies that the local sequence context predominantly dictates the amino acid interactions that can take place, making IDPs more amendable to prediction of their characteristics from sequence. Throughout the last decade many disorder prediction algorithms were designed to exploit the information contained within the amino acid sequence of an IDP; there are more than 50 disorder predictors worldwide (He et al., 2009). The first disorder predictors, such as DisEMBL (Linding et al., 2003) were primarily based on the distinct compositional bias of IDPs. They were followed by faster and more reliable algorithms, such as IUPred (Dosztanyi et al., 2005), RONN (Yang et al., 2005), and Espritz (Walsh et al., 2012). Some of these more advanced methods rely on machine learning techniques (Bellay et al., 2012), or combine the results of several algorithms, such as the meta-predictor metaPrDOS (Ishida and Kinoshita, 2008). Overall, the accuracy of most predictors is consistently above 80%, with the best methods currently peaking around 85% (Monastyrskyy et al., 2014). Alternatively, the novel Dynamine approach predicts backbone dynamics, which correlates (negatively) with intrinsic disorder (Cilia et al., 2014); interestingly, this approach is trained on estimations directly from NMR data and avoids structure-based information, complex machine-learning and evolutionary information (Cilia et al., 2013). The distribution of charged amino acids in the sequence of an IDP can also offer information on whether the protein chain is extended or collapsed (Das and Pappu, 2013).

Often it is unnecessary even to predict disorder, since there are a number of openly accessible online resources that store information of the disorder content of specific proteins. The Disordered Protein Database (DisProt) is the primary one of these sequence-based resources (Sickmeier et al., 2007). DisProt is manually curated, and stores information on proteins for which intrinsic disorder was experimentally determined. Where available, the proteins are also annotated with their known functions. However, DisProt houses data for 694 disordered proteins, which is only a minor fraction of the expected number of IDPs. MobiDB (Potenza et al., 2015) and D2P2 (Oates et al., 2013) on the other hand are online resources that store IDPs identified using prediction algorithms from the whole UniProt in addition to experimentally determined ones.

Sequence information on intrinsic disorder can be exploited for more than merely the prediction of disordered residues. In fact, there are many recent algorithms that aim at predicting functional sites and/or the functional role of IDRs. For example, larger hydrophobic residues such as tryptophan and leucine are often found within peptide motifs that act as recognition units located within IDR segments, called molecular recognition features (MoRFs) (Mohan et al., 2006; Fuxreiter et al., 2007; Brown et al., 2010). Disordered motifs are generally short, 3-15 residue long segments; therefore identifying them poses a computational challenge (Gould et al., 2010). Consequently, predicting functional sites in IDRs is not straightforward, and prone to high false positive rates (Tompa, 2011). Additional layers of information can enhance the performance, for example MoRFpred, which uses order/disorder patterns (Cheng et al., 2007), ANCHOR, which estimates the interaction of a segment with a general partner (Meszaros et al., 2009) or DisCons, which takes into consideration the evolutionary conservation of both the amino acid sequence and of the disorder as a feature (Varadi et al., 2015).

## Structural representation of IDPs

Ideally it would be possible to describe the structure of an IDP/IDR in full atomic detail. Indeed, the set of conformations IDPs sample is often not completely random, for example both p21 and p27 are known to sample distinct secondary structural elements that are biologically relevant and involved in conformation selection (Kriwacki et al., 1996; Sivakolundu et al., 2005). Due to their inherent conformational flexibility, however, the structure of an IDP cannot be described with a single, static conformation (Tompa and Varadi, 2014). This conformational diversity of IDPs precludes crystallization and examination by X-ray crystallography is therefore not a viable option. NMR spectroscopy, while more attuned to conformational diversity, remains hindered by distinct difficulties such as peak overlap (Bellay et al., 2012), while traditional structure calculation protocols do not properly account for multiple conformations (Vranken, 2014).

**Figure 1:**
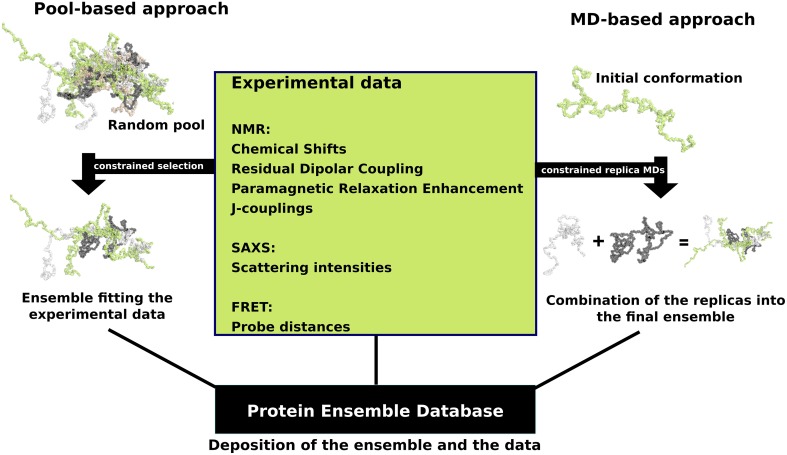
**Schematic view of the two main ensemble modeling approaches**. Pool-based ensemble modeling (left) starts by generating a pool of random or semi-random conformations based on the protein sequence. Subsets of conformations are selected iteratively from the pool and theoretical parameters are calculated for each conformer in the subset. The final ensemble consists of conformations for which the theoretical parameters are in agreement with the experimental data. MD-based approaches start by initiating short replica MD simulations in parallel using an initial conformation. The MD replicas are constrained with the experimental data. The final ensemble is a combination of the resulting replica runs.

In response to this challenge, a number of approaches were developed that combine experimental data with computational methodology with the aim to accurately describe the full conformational ensemble adopted by IDPs. Experimental data from techniques that rely on measurements performed in solution are particularly well suited for studying the dynamic structure of an IDP, even though they often represent an average over the different conformations that are adopted by the IDP. These experimental measurements predominantly include NMR-derived parameters, such as chemical shifts (CSs) (Jensen et al., 2011), residual dipolar couplings (RDCs) (Mittag et al., 2010b), paramagnetic relaxation enhancements (PREs) (Mittag et al., 2010b), and J-couplings (Mittag et al., 2010b), as well as scattering intensities from small-angle X-ray scattering (SAXS) (Allison et al., 2009) and probe distances from Forster resonance energy transfer (FRET) (Haas, 2012). These experimental data are then combined with computational methods to determine an ensemble of conformations for an IDP, with two main approaches being used; the first approach is referred to as pool-based modeling, while the second one is based on molecular dynamics (MD) simulations (Tompa and Varadi, 2014) (Figure 1).

When using a pool-based approach, such as the ensemble optimization method (EOM) (Bernado et al., 2007) which was designed to model ensembles based on SAXS data, the initial step is to generate a very large random or semi-random pool of conformations based on the amino acid sequence of the IDP/IDR with algorithms such as Flexible-Meccano (Ozenne et al., 2012). The sampling of conformations can be biased using experimental data, such as secondary structure propensities derived from chemical shifts. Next, theoretical parameters are estimated for each conformer in the pool, for example the theoretical scattering intensities calculated by software such as CRYSOL (Bernado and Svergun, 2012). Selection algorithms are then deployed in order to select subsets of the pool in a way that when the theoretical parameters are averaged over a subset, they are in excellent agreement with the experimental data (Sibille and Bernado, 2012). Another example is the ENSEMBLE methodology, which uses as input a large set of conformations together with relevant experimental data, and then prunes the ensemble of conformations to a smaller subset. During the filtering step, conformations are assigned weights so that the resulting ensemble average values fit the experimental input values. Structures that do not contribute to this fitting are discarded (Krzeminski et al., 2013).

In contrast, ensemble modeling procedures are based on molecular dynamics (MD) simulations and begin with random conformations in parallel. Multiple “replica” simulations are initiated using these initial conformations, and constraints are applied over multiple models based on the experimental data, i.e., sets of conformations are required to satisfy experimentally determined constraints, such as pair-wise distances or secondary structure propensities (Cavalli et al., 2013). Given the conformational heterogeneity of IDPs, which sample an extensive range of conformations during their biological lifetime, extensive simulations are required to ensure adequate sampling of relevant regions of the conformational space. This significantly increases the computational costs associated with IDP simulations and makes it difficult to achieve even for systems of modest size. Recent developments to address these issues include techniques such as multi-scale enhanced sampling (MSES) (Lee and Chen, 2015) and replica exchange with guided annealing (RE-GA) (Zhang and Chen, 2014). The MSES protocol combines coarse-grained, topology-based models with atomistic force fields to enhance sampling and was recently optimized for simulating IDP conformational ensembles, where it could capture reversible helix-coil transitions (Lee and Chen, 2015). RE-GA has been suggested to be suitable for systems with small conformational transition barriers (as is the case for IDPs), and helped the disordered kinase inducible domain (KID) protein to efficiently escape non-specific compact states while requiring less computation (Zhang and Chen, 2014).

Therefore, the main differences between these two classes of modeling techniques are that the pool-based approach is much faster; conformations can be easily generated, but the final results strongly depend on the quality and diversity of this initial pool of conformations. The ensemble modeling MD approaches are in contrast very slow; conformational sampling depends on the MD simulation, but they have the advantage that the experimental data are continuously applied, a timeline of conformational changes is available, and due to their more rigorous simulation of physical reality, they should give a better representation of the thermally accessible structural ensemble.

These two modeling approaches (i.e., pool and MD-based) are currently the state-of-the-art and have been applied to generate the structural ensemble of many IDPs (Table 1). These ensemble models are, however, not straightforward to interpret (Tompa and Varadi, 2014). The key issue is that the experimental information that is available to either filter or constrain during the calculation is very sparse compared to the immense degree of conformational freedom the IDP experiences in solution, resulting in a hugely underdetermined problem. As a direct consequence, many ensembles of models can describe the experimental data equally well, allowing multiple, ambiguous solutions that strongly depend on the calculation approach and the amount and type of experimental data available. In fact, one can model the ensemble of an IDP with an excellent fit to the data, then discard the ensemble and remodel another, unique and different ensemble with an equally good fit. In this sense, ensembles should be considered as a whole and their structural characteristics analyzed as the average over the ensemble, and over-interpretation of single conformations should be avoided. However, if certain characteristics or pre-formed secondary structural elements are consistently modeled in multiple ensembles, then such structural features might be functionally relevant.

**Table 1:** **Recently published ensemble models from the Protein Ensemble Database**.

**Protein**	**Data type**	**Protocol**	**PED ID**	**References**
Sic1/Cdc4	NMR and SAXS	Pool-based	PED9AAA	Mittag et al., 2010b
p15 PAF	NMR and SAXS	Pool-based	PED6AAA	De Biasio et al., 2014
MKK7	NMR	Pool-based	PED5AAB	Kragelj et al., 2015
Beta-synuclein	NMR	MD-based	PED1AAD	Allison et al., 2014
P27 KID	NMR	MD-based	PED2AAA	Sivakolundu et al., 2005

The field of ensemble modeling therefore still presents exciting opportunities for further development, and several important issues will have to be addressed before the techniques become more reliable. In our view, the first is increasing the number of experimentally derived constraints, which will lead to higher quality models, or cross-validation with new types of experimental data, which will also increase the power of ensembles. The second is the further incorporation of knowledge based information into the calculations, such as the results from reliable predictions or improved force fields. The third is that specific validation and evaluation approaches are required for these ensembles, likely starting from the currently well-developed NMR validation field (Rosato et al., 2013; Vuister et al., 2014) but with better accounting for multiple conformations (Vranken, 2014). Especially NMR CS values, whenever available, are very useful for the estimation of residue-level backbone and side-chain dynamics (Berjanskii and Wishart, 2005, 2013) as well as secondary structure populations (Shen and Sali, 2006; Camilloni et al., 2012), with new methods providing reference chemical shift values for IDPs (Tamiola et al., 2010). They are already effectively used to generate pools with predetermined conformations and as restraints in molecular dynamics simulations (Krieger et al., 2014), but have immense potential for the validation and evaluation of ensembles. Finally, the overwhelming majority of the already generated ensemble models were previously unavailable to the scientific community, impeding the establishment of standardized validation and evaluation protocol. The Protein Ensemble Database (PED) is an international initiative launched to address this issue, effectively making the experimental data and the ensemble models available to the scientific community (Varadi et al., 2014). This is expected to facilitate the development of the next generation of ensemble modeling techniques, and should provide a basis for defining standards of validation and evaluation.

## Toward the functional interpretation of IDP ensembles

While ensemble models do not yet possess a predictive power comparable to that of the structure of folded proteins/domains, these models can already offer insights regarding the function of an IDP. Through the integration of experimental data into an ensemble model, functionally important segments might be inferred. For example, transient secondary structural elements in the ensemble of an IDP are often important in terms of function. Such pre-formed elements are often molecular recognition units, playing major roles in binding to various partners. For example, thep27 protein samples transient helices are consistent with the secondary structure of the bound state p27-Cdk2-cyclin (Sivakolundu et al., 2005). Therefore, in accord with the notion of conformational selection, if a certain secondary structural element is sampled in the ensemble, it might be functionally relevant in the bound form as well (Yoon et al., 2012). Again, such interpretations have to be treated with caution given that the ensembles are based on lower resolution, averaged experimental observations, and the ensemble models should therefore be accurate on average, not by single conformations.

## Conclusions

IDPs are involved ubiquitously in biological processes, and play essential roles in the regulation of complex cellular systems (Tompa et al., 2006; Dunker et al., 2015). These multi-purpose proteins combine conformational flexibility with an enrichment of binding motifs and post-translational modification sites (Iakoucheva et al., 2004; Wang et al., 2011; Hsu et al., 2013). Due to their biological importance, it is imperative to characterize them and attempt to relate their sequence and structure with their physiological roles (Tompa, 2011). Such an endeavor has the potential to offer valuable insights that can be translated into new drugs and therapies (Funk and Galloway, 1998; Metallo, 2010; Rezaei-Ghaleh et al., 2012). Sequence-based *in silico* techniques such as disorder prediction algorithms are already comparable in terms of accuracy to that of the secondary structure prediction algorithms of folded proteins (Monastyrskyy et al., 2014), and functional prediction algorithms are also widely available (Cheng et al., 2007; Meszaros et al., 2009; Varadi et al., 2015). Yet, a major breakthrough is expected when ensemble models based on diverse experimental data prove to be biologically relevant, so that we can confidently infer specific protein function from the structural representation of an IDP (Tompa and Varadi, 2014). However, in order to realize this goal a number of challenges need to be tackled first (Tompa, 2011). Chief among these issues are improving the amount and available types of experimental data and establishing standardized protocols for the validation and evaluation of the ensemble modeling procedures. Only then can the field advance in terms of increasing the predictive power of ensemble models (Tompa and Varadi, 2014).

## Conflict of interest statement

The authors declare that the research was conducted in the absence of any commercial or financial relationships that could be construed as a potential conflict of interest.
